# Gemin6 promotes c‐Myc stabilisation and non‐small cell lung cancer progression via accelerating AURKB mRNA maturation

**DOI:** 10.1002/ctm2.811

**Published:** 2022-04-22

**Authors:** Jie Lin, Baiyang Liu, Yong Zhang, Li Lv, Dating Cheng, Wenhui Zhang, Yulin Shi, Xiulin Jiang, Lin Tang, Yixiao Yuan, Haoqing Zhai, Qiushuo Shen, Qiuxia Xiong, Zhixian Jin, Yongbin Chen, Cuiping Yang

**Affiliations:** ^1^ The Second Affiliated Hospital of Kunming Medical University Kunming China; ^2^ Key Laboratory of Animal Models and Human Disease Mechanisms of Chinese Academy of Sciences & Yunnan Province Kunming Institute of Zoology Kunming Yunnan China; ^3^ Kunming College of Life Science University of Chinese Academy of Sciences Beijing China; ^4^ Department of Pathology Cancer Hospital of China Medical University Shenyang Liaoning China; ^5^ Kunming Medical University Kunming China; ^6^ The International Peace Maternity and Child Health Hospital School of Medicine Shanghai Jiao Tong University Shanghai China; ^7^ Shanghai Key Laboratory of Embryo Original Diseases Shanghai China

To the Editor:

Lung cancer is the leading cause of cancer death worldwide, and around 85% of patients are grouped into non‐small cell lung cancer (NSCLC) based on the histological characteristics.^1^ The survival of motor neurons (SMN) complex has been demonstrated to play critical roles in the biogenesis of ribonucleoprotein complexes (RNPs), and the reduced expression of the causative gene SMN leads to spinal muscular atrophy (SMA).^2^, ^3^ The SMN complex mainly consists of SMN, Gemin2, 3, 4, 5, 6 and 7. SMN interacts with Gemin2, 3, 5 and 7 directly, whereas Gemin4 and 6 binding to SMN relies on Gemin3 and 7, respectively. In this study, we revealed the oncogenic role of Gemin6/AURKB/c‐Myc axis in lung adenocarcinoma, and the promising anticancer potential of sertindole treatment with probable clinical application in the future.

As the functional roles of SMN complex in NSCLC remain elusive, we examined the expressions of individual components of SMN complex, and found that Gemin6 is unanimously upregulated in multiple types of human cancer, including NSCLC (Figure 1A,B; Figure S1A–C). Gemin6 high expression was identified to correlate with worse clinical outcome (Figure 1C–H; Figure S1D,E; Table S1). Furthermore, the ROC curve analysis of the SMN complex factors showed that Gemin6 exhibits the highest AUC value of 0.936 (Figure 1I). As expected, real‐time RT‐PCR and immunoblot assays validated that Gemin6 was highly expressed in NSCLC tissues and cell lines (Figure 1J–M; Figure S1F,G). We also identified the mutation pattern of Gemin6 in pan‐cancers including NSCLC (Figure S1H; Table S2). Hypomethylation in Gemin6 promoter region was uncovered to be positively associated with its transcript expression level in multiple tumours (Figure 1N; Figure S1I,J). In addition, the decreased expression of Gemin6 in normal control cell line BEAS‐2B and tumour cells could be reversed by DNA methylases inhibitor 5‐azacytidine (5‐Aza) treatment (Figure 1O).

**FIGURE 1:**
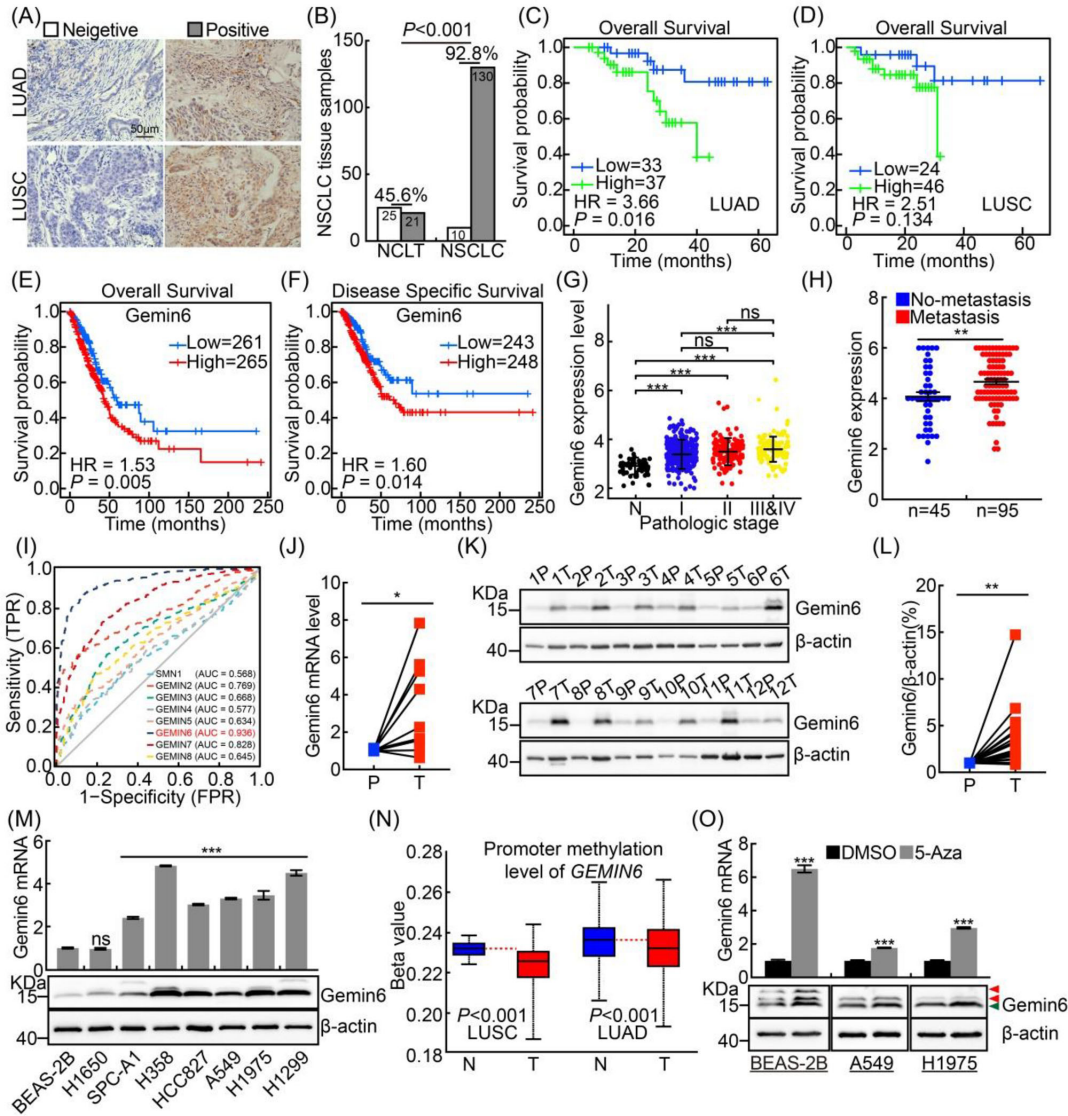
Gemin6 is highly expressed in NSCLC. (A and B) Representative images for Gemin6 IHC staining in tissue sections (A). (B) Quantification data for (A), and chi‐square test was used. NCLT: noncancerous control lung tissues; LUAD: lung adenocarcinoma; LUSC: lung squamous cell carcinoma. Scale bar: 50 μm. (C–F) Gemin6 high expression correlates with worse survival rate; data were collected from NSCLC tissue samples (C: LUAD, D: LUSC) and TCGA database (E and F: LUAD). (G) Gemin6 high expression positively correlates with the stage in NSCLC, data were collected from TCGA database, and unpaired *t*‐test was used. (H) The expression of Gemin6 in NSCLC tissue samples (no metastasis: 45; metastasis: 95). Unpaired *t*‐test. (I) The ROC curve analysis for SMN family members using TCGA database. The AUC (area under the curve) numbers are indicated. (J) The expressions of Gemin6 mRNA in fresh tissues examined by real‐time RT‐PCR. T: tumour; P: paracancerous tissues; *n* = 10; paired *t*‐test. (K and L) The expressions of Gemin6 proteins in fresh tissues examined by immunoblot. (L) Quantification data for (K), *n* = 18. Paired *t*‐test. (M) The mRNA and protein expressions of Gemin6 in indicated cell lines were verified by real‐time RT‐PCR (top) and immunoblot (bottom). Unpaired *t*‐test. (N) The methylation levels in Gemin6 promoter were analysed using UNCLAN database. N: normal; t: tumour. (O) The mRNA and protein expression levels of Gemin6 in indicated cells were examined by real‐time RT‐PCR (top) and immunoblot (bottom), with or without 5‐azacytidine (5‐Aza, 5 μM) treatment for 24 h. Red arrows: non‐specific bands; green arrow: Gemin6. Unpaired *t*‐test. Bars are the mean value ± SEM. *p  < .05, **p  < .01, ***p  < .001. Gem6 = Gemin6

Gemin6 transcript was then inhibited with two lenti‐viral shRNAs in A549 and H1975 (Figure 2A; Figure S2A). We found that Gemin6 knockdown repressed tumour cell proliferation examined by growth curve, BrdU incorporation and colony formation assays (Figure 2B–G; Figure S2B–D). Furthermore, we uncovered that the cell cycle was arrested at G0/G1 phase after Gemin6 knockdown, and the key regulators for G0/G1 cell cycle transition, including CDK2, CDK4 and CDK6 were also markedly reduced upon Gemin6 knockdown (Figure 2H–J; Figure S2E–G). The cell migration ability was also reduced upon Gemin6 knockdown both in vitro and in vivo (Figure 2K–M; Figure S2H–P). In line with the findings in vitro, the xenograft tumour masses, tumour weights and volumes in Gemin6 inhibition groups were markedly impeded compared to the control group (Figure 2N–Q; Figure S2N).

**FIGURE 2:**
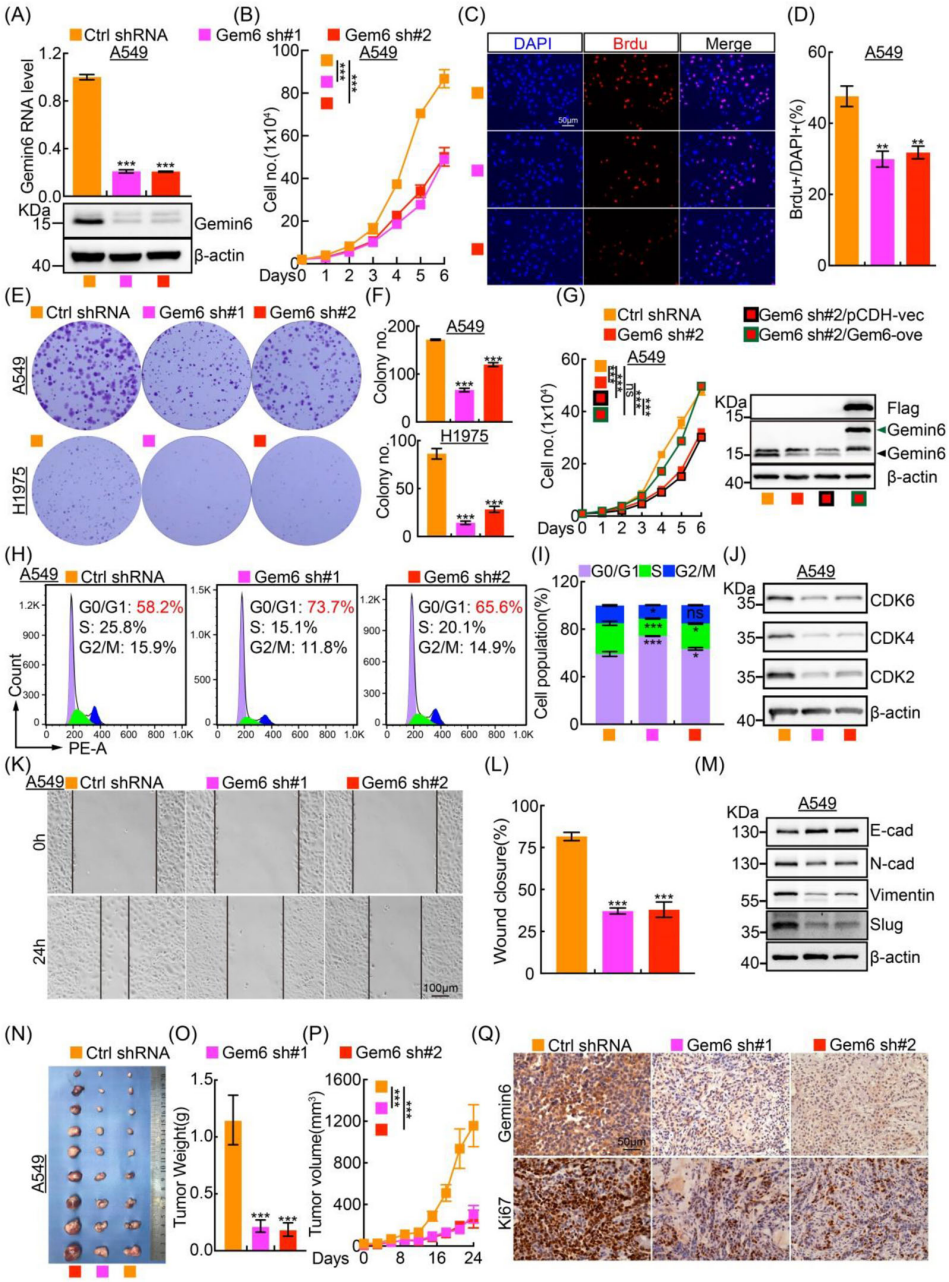
Gemin6 promotes tumour cell proliferation and migration. (A) Establishment of Gemin6 knockdown in A549 verified by real‐time RT‐PCR (top) and immunoblot (bottom). Ctrl = control, sh#1 = shRNA#1, sh#2 = shRNA#2. Unpaired *t*‐test. (B) The growth curve of indicated cells. One‐way ANOVA. (C and D) Representative immunofluorescence staining of BrdU incorporation assay in A549. Scale bar: 50 μm. (D) Quantification data for (C). Unpaired *t*‐test. (E and F) The colony assay for A549 and H1975. (F) Quantification data for (E). Unpaired *t*‐test. (G) The growth curves of indicated cells (left), and the forced expression of Gemin6 was validated by immunoblot (right). Green arrow: exogenous Gemin6‐Flag; black arrow: endogenous Gemin6. One‐way ANOVA. (H and I) Indicated cells were stained by PI, and the cell cycle transition was examined by FACS analysis. (I) Quantification data for (H). Unpaired *t*‐test. (J) Total cell extracts were examined by immunoblot to detect indicated protein expressions with indicated antibodies. (K and L) Representative images for the wound healing assay in A549. Scale bar: 100 μm. (L) Quantification data for (K). Unpaired *t*‐test. (M) Indicated EMT signaling pathway regulators were examined by immunoblot with indicated antibodies. (N) Representative xenograft tumours generated using A549 cells are shown. (O) Statistical histogram of the weights of xenograft tumours. Unpaired *t*‐test, *n* = 9. (P) Statistic changes of tumour volumes at different time points are shown. One‐way ANOVA, *n* = 9. (Q) Representative IHC staining images of Gemin6 and Ki67 in the xenograft tumours. Scale bar: 50 μm. Bars are the mean value ± SEM. *p  < .05, **p  < .01, ***p  < .001. ns = no significant difference

To decipher the underlying mechanism by which Gemin6 regulates NSCLC progression, we found that Gemin6 was involved in c‐Myc, but not E2F, related signaling pathway (Figure 3A,B; Figure S3A). However, the reduced c‐Myc proteins, but not the transcripts, in Gemin6 knockdown groups were detected compared to control (Figure 3C–F; Figure S3B–E). As expected, Gemin6 knockdown‐reduced cell proliferation ability could be overcome by c‐Myc overexpression (Figure 3G). These findings prompted us to hypothesise that Gemin6 regulates the post‐translational modification of c‐Myc proteins. As documented by other studies, c‐Myc proteins could be stabilised by deubiquitinases USP36/USP28,^4^, ^5^ or degraded by E3 ligase FBXW7/SKP2.^6^, ^7^ To our surprise, the above c‐Myc regulators’ mRNA expressions were not markedly deregulated upon Gemin6 inhibition (Figure 3H; Figure S3F,G). However, recent findings identified that phosphorylation of c‐Myc Serine 67 site mediated by AURKB, prevents its degradation in a proteasome signaling pathway‐dependent manner,^8^ which was uncovered to be significantly reduced upon Gemin6 knockdown (Figure 3I; Figure S3H). Furthermore, we found that Gemin6 expression positively correlates with AURKB, and the mRNA and protein expressions of AURKB were both decreased in Gemin6 knockdown cells (Figure 3J,K; Figure S3I). In addition, we showed that AURKB overexpression reversed Gemin6 knockdown‐reduced cell proliferation and migration abilities (Figure 3L–Q). In line with the findings that SMN complex regulates the biogenesis of RNPs, we revealed that the SMN complex formation, the maturation process, but not the stability of AURKB mRNA, were decreased upon Gemin6 knockdown (Figure 3R–T; Figure S3J–L). As expected, we showed that AURKB serves as a prognostic biomarker and is highly expressed in NSCLC, which correlates with worse overall survival (OS) and disease‐specific survival (DSS) rates in LUAD (Figure 3U,V; Figure S3M–P).

**FIGURE 3:**
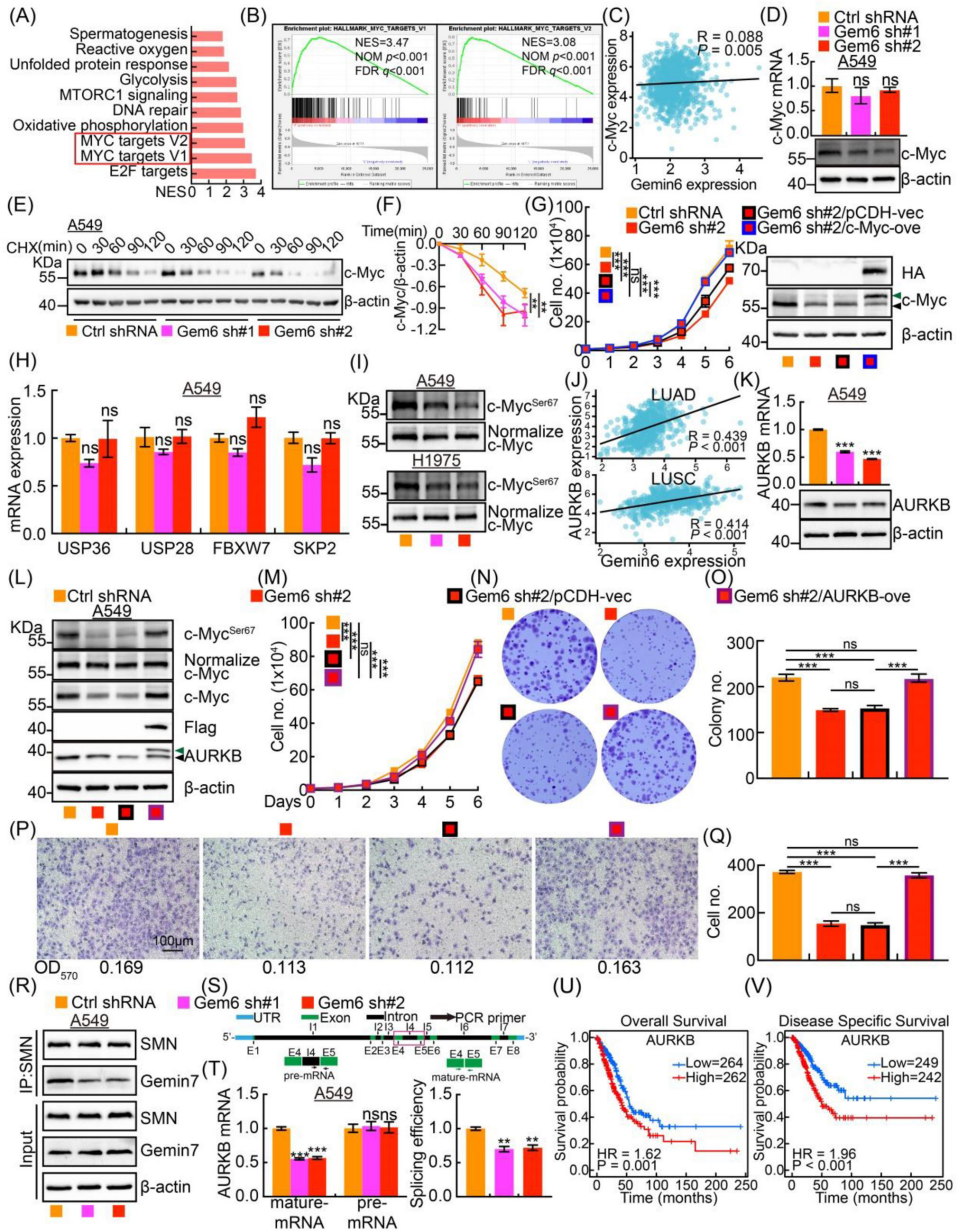
Gemin6 stabilises c‐Myc proteins. (A) The gene set enrichment analysis (GESA) result is shown. (B) Gemin6 was involved in c‐Myc‐related signaling pathways. (C) The mRNA expression correlation between c‐Myc and Gemin6 in NSCLC. Data from TCGA database. (D) c‐Myc expression levels in A549 were detected by real‐time RT‐PCR (top) and immunoblot (bottom). Unpaired *t*‐test. (E and F) Indicated A549 cells were treated with CHX (100 μg/ml), and c‐Myc proteins were examined at indicated time point. (F) Quantification data for (E). One‐way ANOVA. (G) The growth curves for indicated cells (left). Forced expression of c‐Myc was verified by immunoblot using indicated antibodies (right). Green arrow: exogenous c‐Myc‐HA; black arrow: endogenous c‐Myc. (H) The relative mRNA expressions of indicated genes important for c‐Myc protein stabilisation examined by real‐time RT‐PCR. Unpaired *t*‐test. (I) Indicated protein expressions were detected by immunoblot with indicated antibodies in A549 and H1975. In order to detect the phosphorylation level change, the total c‐Myc proteins were normalised. (J) The mRNA correlation between AURKB and Gemin6 in LUAD and LUSC. Data from TCGA database. (K) The relative AURKB expressions were verified by real‐time RT‐PCR (top) and immunoblot (bottom) in A549. Unpaired *t*‐test. (L) Forced expression of AURKB was verified by immunoblot using indicated antibodies. Green arrow: exogenous AURKB‐Flag; black arrow: endogenous AURKB. (M) The growth curves for indicated cells. One‐way ANOVA. (N and O) The colony assay for indicated cells. (O) Quantification data for (N). Unpaired *t*‐test. (P and Q) Representative images for the trans‐well assay in indicated cells. Scale bar: 100 μm. The OD_570_ values are shown at the bottom. (Q) Quantification data for (P). Unpaired *t*‐test. (R) Indicated protein expressions were detected by immunoblot with indicated antibodies in indicated cells. (S) Schematic cartoon for the assay verifying AURKB mRNA maturation. Red rectangle indicates the PCR region. (T) Real‐time RT‐PCR result demonstrating AURKB mRNA maturation ratios in A549. Unpaired *t*‐test. (U and V) AURKB high expression correlates with worse OS and DSS rates in LUAD. Bars are the mean value ± SEM. *p  < .05, **p  < .01, ***p  < .001. ns = no significant difference

To further explore the clinical value of Gemin6, we then examined the potential drug repurposing activities of 10 drugs, selectively targeting dopamine or serotonin receptors from FDA‐Approved Drug Library Mini, in NSCLC by blocking Gemin6 expression (Table S3).^9^ Compound sertindole was identified as the only one with the activity decreasing Gemin6 proteins to less than 70% of control level (Figure 4A; Figure S4A). Sertindole has been previously identified as an antagonist targeting dopamine D2 receptors, serotonin 5HT_2A_ receptors and α_1_‐adrenoceptors, which is mainly produced in the central nervous system and gastrointestinal tract.^10^ The cell viability after sertindole treatment was examined, and more dramatic inhibitory effect on tumour cell (A549 and H1975) survival was detected (Figure 4B). Furthermore, we found that sertindole treatment decreased the mRNA expressions of Gemin6 and AURKB, but not c‐Myc (Figure 4C,D; Figure S4B). The cell proliferation and cell migration abilities were also repressed after sertindole treatment, which were markedly reversed by Gemin6 or c‐Myc forced expression, respectively (Figure 4E–I; Figure S4C–I). As expected, the xenograft tumour masses, tumour weights and volumes in sertindole treatment group were markedly inhibited compared to control group, evidenced by deceased Ki67, Gemin6 and c‐Myc IHC‐positive signals (Figure 4J–O).

**FIGURE 4:**
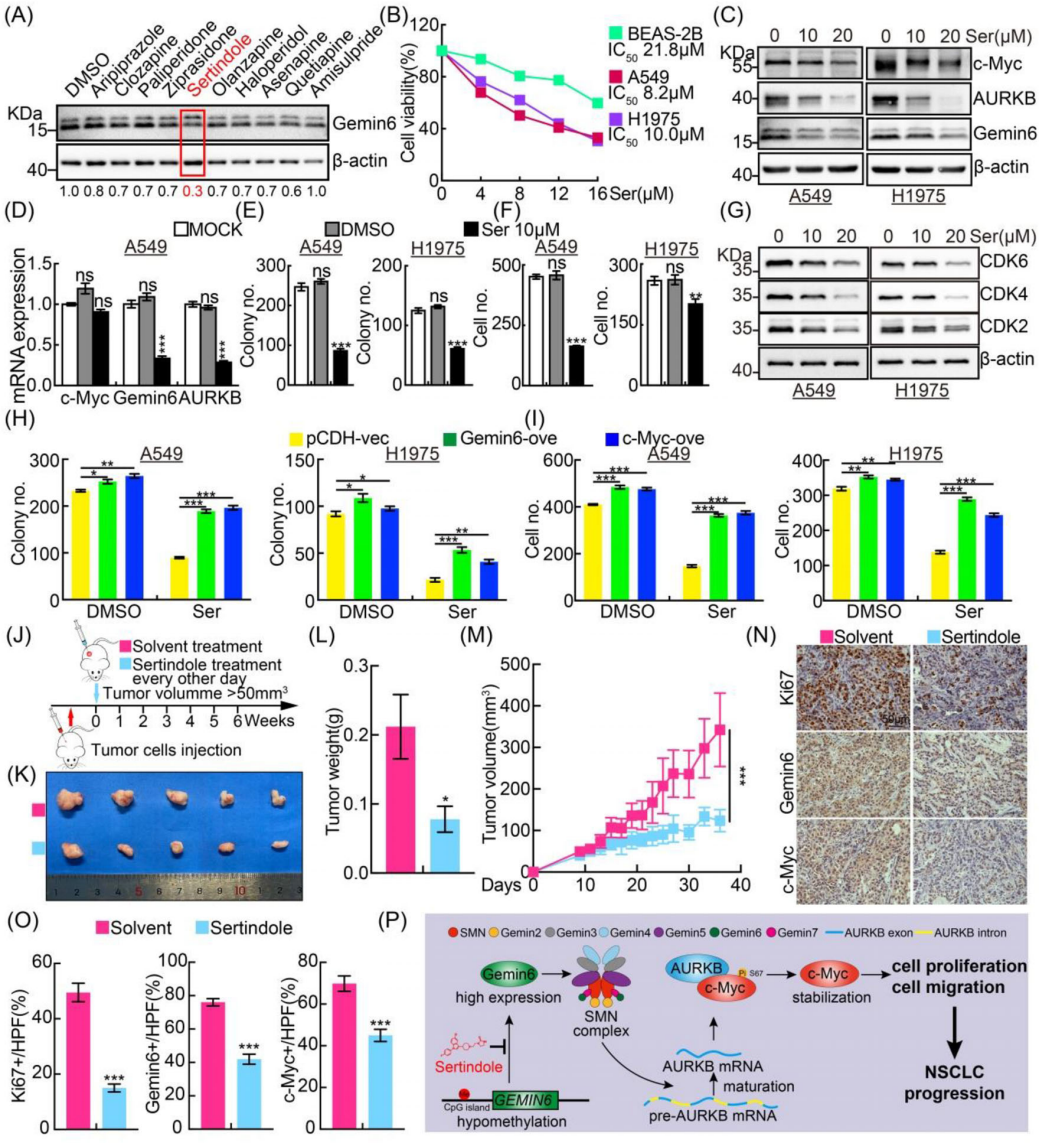
Sertindole acts as a potential antagonist for Gemin6. (A) Drug screen was performed to identify potential drugs repressing Genmin6 protein expression using immunoblot in A549. Cells were incubated with indicated drugs (20 μM) for 48 h before collection. DMSO was used as control. The quantification data for the immunoblot ratio: Gemin6/β‐actin was also shown at the bottom. (B) IC_50_ after sertindole treatment in indicated cell lines. Ser = sertindole. (C) The protein expressions of c‐Myc, Gemin6 and AURKB in A549 and H1975 after 10 or 20 μM sertindole treatment for 48 h were detected by immunoblot with indicated antibodies. (D) The relative mRNA expressions of c‐Myc, Gemin6 and AURKB in A549 with or without 10 μM sertindole treatment for 48 h were detected by real‐time RT‐PCR. Unpaired *t*‐test. (E and F) Sertindole (10 μM) treatment in A549 and H1975 cells decreased cell proliferation and migration examined by colony formation (E) and Transwell (F) assays. Quantification data are shown. Unpaired *t*‐test. (G) The protein expressions of CDK2, CDK4 and CDK6 in A549 and H1975 after sertindole treatment (10 or 20 μM, 48 h), detected by immunoblot. (H and I) Gemin6 or c‐Myc overexpression markedly, but not completely, reversed sertindole treatment (10 μM) reduced cell proliferation and migration abilities by colony formation (H) and trans‐well (I) assays. Quantification data are shown. Unpaired *t*‐test. (J–O) Schematic view of xenograft mouse model treated with solvent or sertindole (J). A549 wild‐type cells were injected. Representative xenograft tumour mass images (K), tumour weight (L) and tumour volumes (M), are shown for indicated groups. Unpaired *t*‐test and one‐way ANOVA, *n* = 5. (N) Representative IHC staining images of Ki67, Gemin6 and c‐Myc in indicated xenograft tumour sections. (O) Quantification data for (N). Unpaired *t*‐test. Scale bar: 50 μm. (P) The working model for Gemin6 in NSCLC. Gemin6 is increased in NSCLC due to its promoter hypomethylation, which accelerates the mRNA maturation process of AURKB. High expression of AURKB promotes oncoprotein c‐Myc phosphorylation and stabilisation, leading to increased tumour cell proliferation, migration and then tumour progression. Sertindole was identified as a potent antagonist for Gemin6, which inhibits tumour growth both in vitro and in vivo. Bars are the mean value ± SEM. *p  < .05, **p  < .01, ***p  < .001. ns = no significant difference

We observed that AURKB mRNA was reduced upon Gemin6 inhibition. However, how Gemin6 acts alone or cooperates with other SMN complex components to regulate AURKB mRNA splicing or transcription is still unclear. Therefore, it will be necessary to examine the integrity of RNPs or SMN complex upon Gemin6 knockdown in the future. We showed that sertindole, previous identified antagonist targeting dopamine D2 receptors, serotonin 5HT_2A_ receptors and α_1_‐adrenoceptors, inhibited NSCLC progression by inhibiting c‐Myc, who lies at the crossroads of these signaling pathways, and reveals the promising anticancer potentials of sertindole against NSCLC even with lung‐to‐brain metastases in the future (Figure 4P).

## CONFLICT OF INTEREST

The authors declare that there is no conflict of interest.

## Supporting information

Supporting Information
