# Supplementary material for: Gemin6 promotes c‐Myc stabilisation and non‐small cell lung cancer progression via accelerating AURKB mRNA maturation
Source: Clin Transl Med. 2022 Apr 22;12(4):e811. doi: 10.1002/ctm2.811 (PMC9029014; doi:10.1002/ctm2.811)
Supplement: Supplementary file 1 — Supporting Information [file CTM2-12-e811-s001.doc]

**SUPPLEMENTARY MATERIALS**

**Gemin6 promotes c-Myc stabilization and non-small cell lung cancer progression via accelerating AURKB mRNA maturation**

Jie Lin^1,#,*^, Baiyang Liu^2,3,#^, Yong Zhang^4,#^, Li Lv^1^, Dating Cheng^5^, Wenhui Zhang^1^, Yulin Shi^2^, Xiulin Jiang^2,3^, Lin Tang^5^, Yixiao Yuan^5^, Haoqing Zhai^2,3^, Qiushuo Shen^2^, Qiuxia Xiong^5^, Zhixian Jin^5^, Yongbin Chen^2,3,*^, Cuiping Yang^2,3,6,7,*^

#These authors contribute equally to this work

*Correspondence should be addressed to C.P.Y (email: cuipingyang@sjtu.edu.cn); Y.B.C (email: [ybchen@mail.kiz.ac.cn](mailto:ybchen@mail.kiz.ac.cn)); J. L (email: linjie@kmmu.edu.cn)

**MATERIALS AND METHODS**

**Cell culture**

HEK-293T was purchased from ATCC and cultured in DMEM medium containing 10% fetal bovine serum (FBS) and 1% penicillin/streptomycin. BEAS-2B, H1650, SPCA1, H358, HCC827, H1975, A549 and H1299 were purchased from Cobioer, China with STR document, and were cultured in RPMI-1640 medium (Corning) supplemented with 10% fetal bovine serum (FBS), 1% penicillin/streptomycin. All cells were cultured in a constant temperature incubator containing 5% CO_2_ at 37 ^o^C. The above cells were validated to be mycoplasma free by RT-PCR.

**Constructs and shRNA lenti-viral preparation**

Two independent shRNAs targeting two different regions of GEMIN6 mRNA were designed and inserted into pLKO.1 vector (Addgene, Cambridge, USA). *GEMIN6* (NM_024775.10) was cloned into pCDH-MSCV-E2F-eGFP lenti-viral vector fused with a 3×Flag at the C-terminus, and c-Myc (NM_001354870.1) was cloned into pCDH-MSCV-E2F-eGFP lenti-viral vector fused with 6×HA at the N-terminus. The lenti-viruses were generated using the calcium phosphate transfection in HEK-293T cells. In brief, HEK-293T supernatants with lenti-viral plasmids transfection were collected at 48 and 72 hours, respectively. The lenti-viral containing supernatants were used to infect indicated cell lines, and stable cell lines were established by puromycine selection. All the sequences of the primers and oligos used in this study were provided in Table S4.

**Cell proliferation, BrdU incorporation and colony formation assays**

For cell proliferation assay, indicated cells were inoculated into 12-well plates (1x10^4^ cells/well), and cell numbers were quantified daily using automatic Cell Analyzer Countstar (Shanghai Ruiyu Biotech). For BrdU incorporation assay, 4x10^4^ indicated cells were cultured in 8-well plates for 24 hours, then incubated with 10 μM BrdU for 20min, followed by detection with primary (Cell Signaling Technology, Cat# 5292s, dilution 1:1000) and secondary antibodies (Abclonal, Cat# 61303, dilution 1:500) according to previous documented protocol by our group^1^. The cell nuclei were stained with DAPI (4',6-diamidino-2-phenylindole). For colony formation assay, indicated cells (600/well) were inoculated into 6-well plates for 2 weeks, and then fixed with 4% PFA, followed by crystal violet staining. The colonies were photographed and quantified using Nikon inverted microscope (Ti-S).

**Cell migration and cell cycle analysis**

For the wound healing assay, indicated cells were cultured in 6-well plates at the density of 1x10^6^ cells per well. At indicated time point (18~24 hours later), the cells were scratched with sterile tips, and the wound healing abilities were pictured using Nikon inverted microscope (Ti-S), the wound healing area percentage compared to the starting point (%) were quantified. For trans-well assay, 100µL serum-free medium containing 1x10^5^ indicated cells was added into each well (Corning Life Sciences, Cat. 3422), and 600µL complete medium containing 10% FBS was added below each well. After 24 hours of culture and wash, migrated cells were fixed with 4% PFA and stained with crystal violet. The images were photographed using Nikon inverted microscope (Ti-S), then 600µL 33% acetic acid was added and OD_570_ value was measured by spectrophotometer. For cell cycle transition analysis, indicated cells were digested and washed with PBS, fixed with 75% alcohol and then stained with propidium iodide (PI), and different cell populations were examined by FACSAria SORP machine (BD, USA). We used the Annexin V FITC Apoptosis Detection Kit I (556547, BD, China) to examine the cellular apoptosis following the manufacture’s standard protocol.

**Immunoblot and Real-time RT-PCR experiments**

Indicated total proteins were extracted from indicated tissues or cells using RIPA lysis buffer (1mM NaF, 50 mM Tris-HCl, pH 8.0, 120 mM NaCl, 0.5% NP40, 1 mM EDTA), with complete protease inhibitor cocktail (Complete Mini, Roche). The cell lysates were centrifuged and the supernatants were treated at 100℃ for 5~10min, total proteins were transferred to the polyvinylidene ﬂuoride membrane (Millipore) by gel electrophoresis. The total RNAs from indicated cells were extracted using RNAiso Plus Kit (Takara, Cat# 108-95-2). Indicated cells were treated with 100 μg/mL cycloheximide (CHX) or 20 μM MG132 for 24 h. For Real-time RT-PCR assay, indicated cells were lysed by RNAiso Plus. Indicated cells were treated with 1 μg/mL Actinomycin D for time gradient. The total RNAs were reverse transcribed into cDNAs using the RT reagent Kit (Takara Bio, Beijing, Cat# RR047A, China; TIANGEN Biotech, Cat# KR211-02, China). Real-time RT-PCR was performed by FastStart Universal SYBR Green Master Mix (Roche, Cat# 04194194001; TIANGEN Biotech, Cat# FP411-02, China) by Applied Biosystems 7500 machine. All the antibodies used in this study were shown in Table S4.

**Methylation specific PCR (MSP)**

Genomic DNA was extracted from fresh clinical tissue samples by multisource genomic DNA miniprep kit (AXYGEN, Cat# 140). Subsequently, 1µg of DNA treated with DNA Bisulfite conversion kit (TIANGEN, Cat# DP215-02) was detected methylation products by PCR and gel electrophoresis (M: Methylated band, U: Unmethylated band). The Methylation specific PCR primer was designed by MethPrimer 2.0 ^2^.

**Xenograft tumor formation assay**

The 4-5 weeks old male nude mice (Purchased from Vital River Laboratories, Beijing.) were kept in a SPF environment, and the protocols were pre-approved and conducted under the policy of Animal care and Use Committee at the Kunming Institute of Zoology, CAS. Indicated cells were injected subcutaneously into nude mice. Tumor length (L) and width (W) were measured every other day. At the end of the experiment, the mice were sacrificed and the tumor masses were harvested, weighed and photographed. Tumor volumes were calculated by the formula L × W^2^/2. For the metastatic mouse model, 4-5 weeks old male nude mice were injected with 2 × 10^6^ tumor cells via the tail vein. After 6 weeks, the numbers of metastatic nodules in the lung tissue were quantified. The tumor tissues were fixed with 10% formalin and paraffin embedded for H&E and IHC staining subsequently.

**Immunohistochemical staining (IHC)**

Tissue sections were deparaffinized by xylene and rehydrated with gradient alcohol, are treated with sodium citrate buffer (pH6.0) at 95 °C for 20 minutes. The tissue sections were treated with 3% hydrogen peroxide for 20min and then blocked with 10% goat serum (mix with PBS containing 0.2% Tween 20) for 20min. Indicated primary antibodies (mix with 5% goat serum) were incubated overnight at 4°C. Following washes with PBS, tumor sections were incubated with HRP conjugated indicated secondary antibodies at room temperature for 40 min, and then stained with 3, 3-diaminobenzidine tetrahydrochloride (DAB). Slides containing tumor sections were photographed (Olympus BX43F, Japan), and the pictures were examined by Image-Pro Plus 7.0 software (Media Cybernetics, Inc., Silver Spring, MD, USA). Samples were obtained with informed consent and all protocols were approved by The Second Xiangya Hospital of Central South University Ethics Review Board (Scientific and Research Ethics Committee, S-02/2000). Written informed consent was obtained from all patients (Table S1).  Immunohistochemical staining of clinical sample sections were scored independently by LBY and CDT. The evaluation was based on the staining intensity and positive area. Staining intensity for Gemin6 was scored as 0 (negative), 1 (weak), 2 (moderate), and 3 (strong). Staining positive area was scored as 0 (<10%), 1 (1–25%), 2 (26–50%), 3 (>50%), depending on the percentage of positive-stained cells. An optimal cut-off point was defined as follows: 0–2 was considered as negative expression, while 3–6 was considered as positive expression. Agreement between the two evaluators was 95%.

**Drug Screen**

Converting the existing drugs from one therapeutic area to the treatment of other human diseases, known as “drug repurposing” or “drug repositioning,” has recently been applied to shorten the clinical application time. For example, antipsychotic drugs have been widely used for drug repurposing, especially for the potent anti-cancer properties ^3^. Increasing evidence has shown that dopamine and serotonin are involved in tumorigenesis by regulating angiogenesis and tumor cell proliferation, thus agonist or antagonist targeting to dopamine or serotonin related signaling pathways might provide promising candidate drugs for NSCLC treatment in the future ^4-8^. To further explore the clinical value of Gemin6, we then examined the potential drug repurposing activities of 10 drugs, selectively targeting to dopamine or serotonin receptors from FDA-Approved Drug Library Mini, in NSCLC by blocking Gemin6 expression (Table S3). The drug screen was performed by determining Gemin6 protein expression after 20 μM of candidate drug treatments in A549 compared to control treatment. Compound sertindole was identified as the only one with the activity decreasing Gemin6 proteins to less than 70% of control level, compared to groups treated by other drugs (Figure 4A, S4A).

**Ethics Statement**

Mouse care and treatment was approved by the Animal Care and Use Committee at the Kunming Institute of Zoology, Chinese Academy of Sciences. Human samples were obtained with informed consent and all protocols were approved by The Second Xiangya Hospital of Central South University Ethics Review Board (Scientific and Research Ethics Committee, S-02/2000). Written informed consent was obtained from all patients.

**Bioinformatics analysis**

The data sets used for generation of figures in the current study were available to the public. Online database Gene Set Cancer Analysis (GSCA)^9^, GEPIA^10^, Timer 2.0^11^ and UNCLAN^12^ were used to analyze the expression of target genes. The TCGA datasets were used for survival curve, molecular correlation analysis and KEGG pathway enrichment ^13,14^, the KEGG pathway enrichment analysis was performed using the GSEA software ^15^. The significance of the data between two experimental groups was determined by either unpaired or paired two-tailed Student’s *t*-test, and multiple group comparisons were analyzed by one-way ANOVA. *P* < 0.05 (*), *P* < 0.01 (**) and *P* < 0.001 (***), were significant. All analyses were performed using GraphPad Prism.

**Abbreviations in this study**

ACC, Adrenocortical carcinoma; BLCA, Bladder Urothelial Carcinoma; BRCA, Breast invasive carcinoma; CESC, Cervical squamous cell carcinoma and endocervical adenocarcinoma; CHOL, Cholangiocarcinoma; COAD, Colon adenocarcinoma; DLBC, Lymphoid Neoplasm Diffuse Large B-cell Lymphoma; ESCA, Esophageal carcinoma; GBM, Glioblastoma multiforme; HNSC, Head and Neck squamous cell carcinoma; KICH, Kidney Chromophobe; KIRC, Kidney renal clear cell carcinoma; KIRP, Kidney renal papillary cell carcinoma; LAML, Acute Myeloid Leukemia; LGG, Brain Lower Grade Glioma; LIHC, Liver hepatocellular carcinoma; LUAD, Lung adenocarcinoma; LUSC, Lung squamous cell carcinoma; MESO, Mesothelioma; OV, Ovarian serous cystadenocarcinoma; PAAD, Pancreatic adenocarcinoma; PCPG, Pheochromocytoma and Paraganglioma; PRAD, Prostate adenocarcinoma; READ, Rectum adenocarcinoma; SARC, Sarcoma; SKCM, Skin Cutaneous Melanoma; STAD, Stomach adenocarcinoma; TGCT, Testicular Germ Cell Tumors; THCA, Thyroid carcinoma; THYM, Thymoma; UCEC, Uterine Corpus Endometrial Carcinoma; UCS, Uterine Carcinosarcoma; UVM, Uveal Melanoma.

**REFERENCES**

1. Xu P, Jiang L, Yang Y, et al. PAQR4 promotes chemoresistance in non-small cell lung cancer through inhibiting Nrf2 protein degradation. *Theranostics*. 2020;10(8):3767-3778. doi:10.7150/thno.43142

2. Li LC, Dahiya R. MethPrimer: designing primers for methylation PCRs. *Bioinformatics*. Nov 2002;18(11):1427-31. doi:10.1093/bioinformatics/18.11.1427

3. Huang J, Zhao DW, Liu ZX, Liu FK. Repurposing psychiatric drugs as anti-cancer agents. *Cancer letters*. 2018;419:257-265. doi:10.1016/j.canlet.2018.01.058

4. Peters MAM, Walenkamp AME, Kema IP, Meijer C, de Vries EGE, Oosting SF. Dopamine and serotonin regulate tumor behavior by affecting angiogenesis. *Drug Resist Update*. Oct-Dec 2014;17(4-6):96-104. doi:10.1016/j.drup.2014.09.001

5. Chakroborty D, Chowdhury UR, Sarkar C, Baral R, Dasgupta PS, Basu S. Dopamine regulates endothelial progenitor cell mobilization from mouse bone marrow in tumor vascularization. *Journal of Clinical Investigation*. Apr 2008;118(4):1380-1389. doi:10.1172/JCI33125

6. Chakroborty D, Sarkar C, Basu B, Dasgupta PS, Basu S. Catecholamines Regulate Tumor Angiogenesis. *Cancer research*. May 1 2009;69(9):3727-3730. doi:10.1158/0008-5472.CAN-08-4289

7. Moreno-Smith M, Lu CH, Shahzad MMK, et al. Dopamine Blocks Stress-Mediated Ovarian Carcinoma Growth. *Clinical Cancer Research*. Jun 1 2011;17(11):3649-3659. doi:10.1158/1078-0432.CCR-10-2441

8. Nocito A, Dahm F, Jochum W, et al. Serotonin regulates macrophage-mediated angiogenesis in a mouse model of colon cancer allografts. *Cancer research*. Jul 1 2008;68(13):5152-5158. doi:10.1158/0008-5472.CAN-08-0202

9. Liu CJ, Hu FF, Xia MX, Han L, Zhang Q, Guo AY. GSCALite: a web server for gene set cancer analysis. *Bioinformatics*. Nov 1 2018;34(21):3771-3772. doi:10.1093/bioinformatics/bty411

10. Tang Z, Li C, Kang B, Gao G, Li C, Zhang Z. GEPIA: a web server for cancer and normal gene expression profiling and interactive analyses. *Nucleic Acids Res*. Jul 3 2017;45(W1):W98-W102. doi:10.1093/nar/gkx247

11. Li T, Fu J, Zeng Z, et al. TIMER2.0 for analysis of tumor-infiltrating immune cells. *Nucleic Acids Res*. Jul 2 2020;48(W1):W509-W514. doi:10.1093/nar/gkaa407

12. Chandrashekar DS, Bashel B, Balasubramanya SAH, et al. UALCAN: A Portal for Facilitating Tumor Subgroup Gene Expression and Survival Analyses. *Neoplasia*. Aug 2017;19(8):649-658. doi:10.1016/j.neo.2017.05.002

13. Carrot-Zhang J, Chambwe N, Damrauer JS, et al. Comprehensive Analysis of Genetic Ancestry and Its Molecular Correlates in Cancer. *Cancer cell*. May 11 2020;37(5):639-+. doi:10.1016/j.ccell.2020.04.012

14. Liu J, Lichtenberg T, Hoadley KA, et al. An Integrated TCGA Pan-Cancer Clinical Data Resource to Drive High-Quality Survival Outcome Analytics. *Cell*. Apr 5 2018;173(2):400-416 e11. doi:10.1016/j.cell.2018.02.052

15. Subramanian A, Tamayo P, Mootha VK, et al. Gene set enrichment analysis: a knowledge-based approach for interpreting genome-wide expression profiles. *Proceedings of the National Academy of Sciences of the United States of America*. Oct 25 2005;102(43):15545-50. doi:10.1073/pnas.0506580102


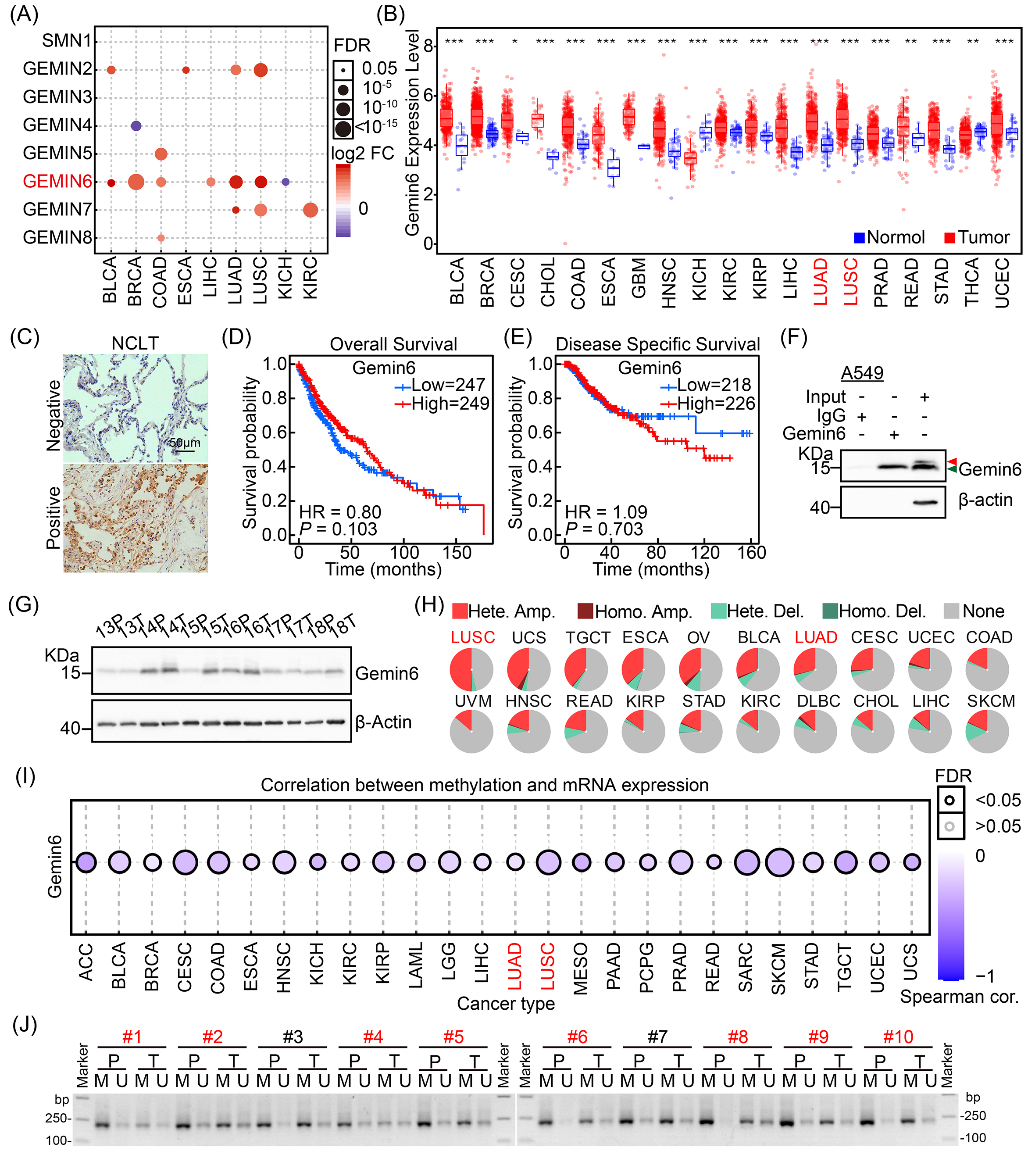


Supplementary Figure S1. Gemin6 is increased in pan-cancers including NSCLC. (A) The expressions of individual component of SMN complex were examined by Gene Set Cancer Analysis (GSCA). (B) The expressions of Gemin6 in various types of human cancer were examined by TIMER 2.0 software. (C) The negative and positive expression of Gemin6 protein expression patterns in noncancerous control lung tissues (NCLT). Scale bar: 50 μm. (D-E) Gemin6 does not significantly correlate with the OS (D) and DSS (E) in LUSC. Data was collected from TCGA database. (F) Immunoblot verifying the specificity of Gemin6 antibody. Red arrow: Non-specific band; Green arrow: Gemin6. (G) The expressions of Gemin6 proteins in fresh tissues examined by immunoblot. T: tumor, P: paracancerous tissues. (H) Gemin6 mutation patterns in a variety of cancers were detected by GSCA dataset. Hete. (Heterozygous); Homo. (Homozygous); Amp. (Amplification); Dele. (Deletion) (I) The correlation between promoter methylation and mRNA expression of Gemin6 in a variety of solid tumors was examined by GSCA dataset. (J) The MSP (methylation specific PCR) gel electrophoresis result was generated using the fresh NSCLC clinical tissue samples. The clinical samples marked in red demonstrate that the promoter region of Gemin6 was hypermethylated in the paracancerous tissue.


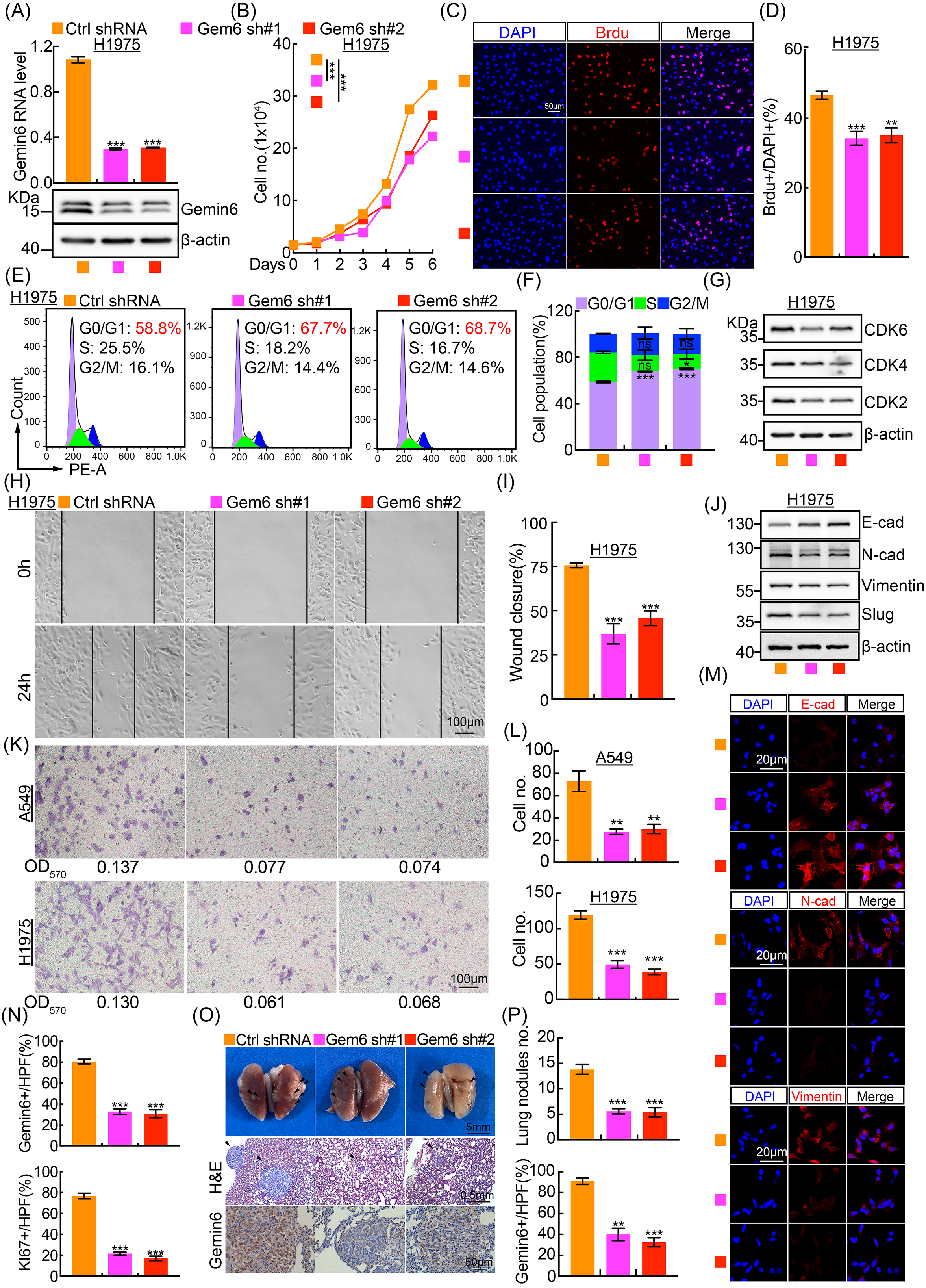
Supplementary Figure S2. Gemin6 promotes tumor cell growth and migration in vitro. (A) Establishment of Gemin6 knockdown in H1975 verified by Real-time RT-PCR (top) and immunoblot (bottom). Ctrl=control, sh#1=shRNA#1, sh#2=shRNA#2. Unpaired *t*-test. (B) The growth curve of indicated cells. One-way ANOVA. (C-D) Representative immunofluorescence staining of BrdU incorporation assay in H1975. Scale bar: 50 μm. (D) Quantification data for (C). Unpaired *t*-test. (E-F) Indicated cells were stained by PI, and the cell cycle transition was examined by FACS analysis. (F) Quantification data for (E). Unpaired *t*-test. (G) Total cell extracts were examined by immunoblot to detect indicated protein expressions with indicated antibodies. (H-I) Representative images for the wound healing assay in H1975. Scale bar: 100 μm. (I) Quantification data for (H). Unpaired *t*-test. (J) Indicated EMT signaling pathway regulators were examined by immunoblot with indicated antibodies. (K-L) Representative images for the trans-well assay in A549 and H1975. Scale bar: 100 μm. The OD_570_ values were shown at the bottom. (L) Quantification data for (K). Unpaired *t*-test. (M) Immunofluorescence images of E-cadherin (E-cad), N-cadherin (N-cad) and Vimentin in A549. (N) Quantification data for Figure 2Q. HPF: high power field. Unpaired *t*-test. (O-P) Representative images for the lung metastasis model in vivo. (P) Quantification data for O. Unpaired *t*-test, n=5. Scale bar: 0.5 mm and 50 μm. Bars are the mean value ± SEM. * *P* < 0.05, ** *P* < 0.01, *** *P* < 0.001, ns = no significant difference.


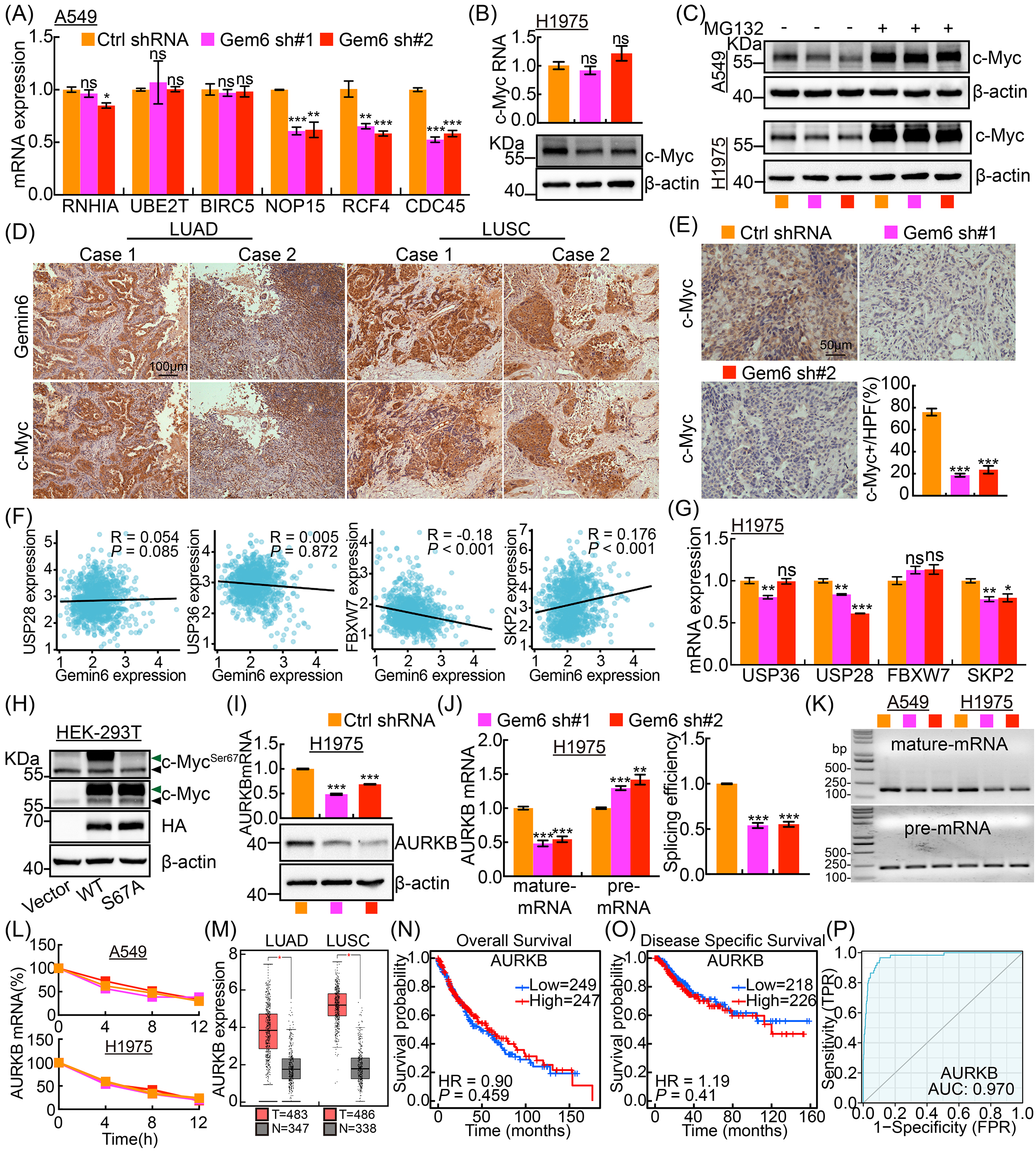
Supplementary Figure S3. Gemin6 is involved in c-Myc signaling pathway. (A) Relative mRNA expressions of indicated genes involved in E2F or c-Myc signaling pathways, examined by Real-time RT-PCR in A549. (E2F targets: RNHIA, UBE2T and BIRC5. MYC targets: NOP15, RCF4 and CDC45), Unpaired *t*-test. (B) The expression levels of c-Myc in indicated H1975 cells were detected by Real-time RT-PCR (top) and immunoblot (bottom). Unpaired *t*-test. (C) Indicated A549 and H1975 cells were treated with MG132 (10 μM) for 6 h before collection. c-Myc proteins were examined by immunoblot. (D) The protein expressions of Gemin6 and c-Myc in NSCLC cancerous tissues examined by IHC. The sections were continuous sections spaced 3µm. Scale bar: 100 μm. (E) Representative IHC staining images of c-Myc using the xenograft tumor sections from Figure 2. Scale bar: 50 μm. HPF: high power field. Unpaired *t*-test. (F) The mRNA expression correlation between indicated genes in NSCLC. Data from TCGA database. (G) Relative mRNA expressions of indicated genes were examined by Real-time RT-PCR. Unpaired *t*-test. (H) The Ser67 phosphorylation specific antibody was validated by immunoblot in HEK-293T cells. Green arrow: exogenous c-Myc; Black arrow: Endogenous c-Myc. Vector: pCDNA3.1, WT: HA-tagged c-Myc, S67A: HA-tagged c-Myc^S67A^. (I) The relative AURKB transcript was examined by Real-time RT-PCR (top) and immunoblot (bottom) in H1975. Unpaired *t*-test. (J) Real-time RT-PCR result demonstrating AURKB mRNA maturation ratios in H1975. Unpaired *t*-test. (K) The gel electrophoresis result was generated using the Real-time RT-PCR products in Figure 3T and S3J. (L) Indicated cells were treated with Actinomycin D (1µg/mL), and the relative mRNA expression level of AURKB were examined at indicated time point. (M) The expression of AURKB in LUAD and LUSC patients were detected by GEPIA database. (N-O) AURKB high expression does not significantly correlate with the OS and DSS rates in LUSC. (P) The ROC curve analysis of AURKB. Data from TCGA-LUAD database. Bars are the mean value ± SEM. * *P* < 0.05, ** *P* < 0.01, *** *P* < 0.001, ns = no significant difference.


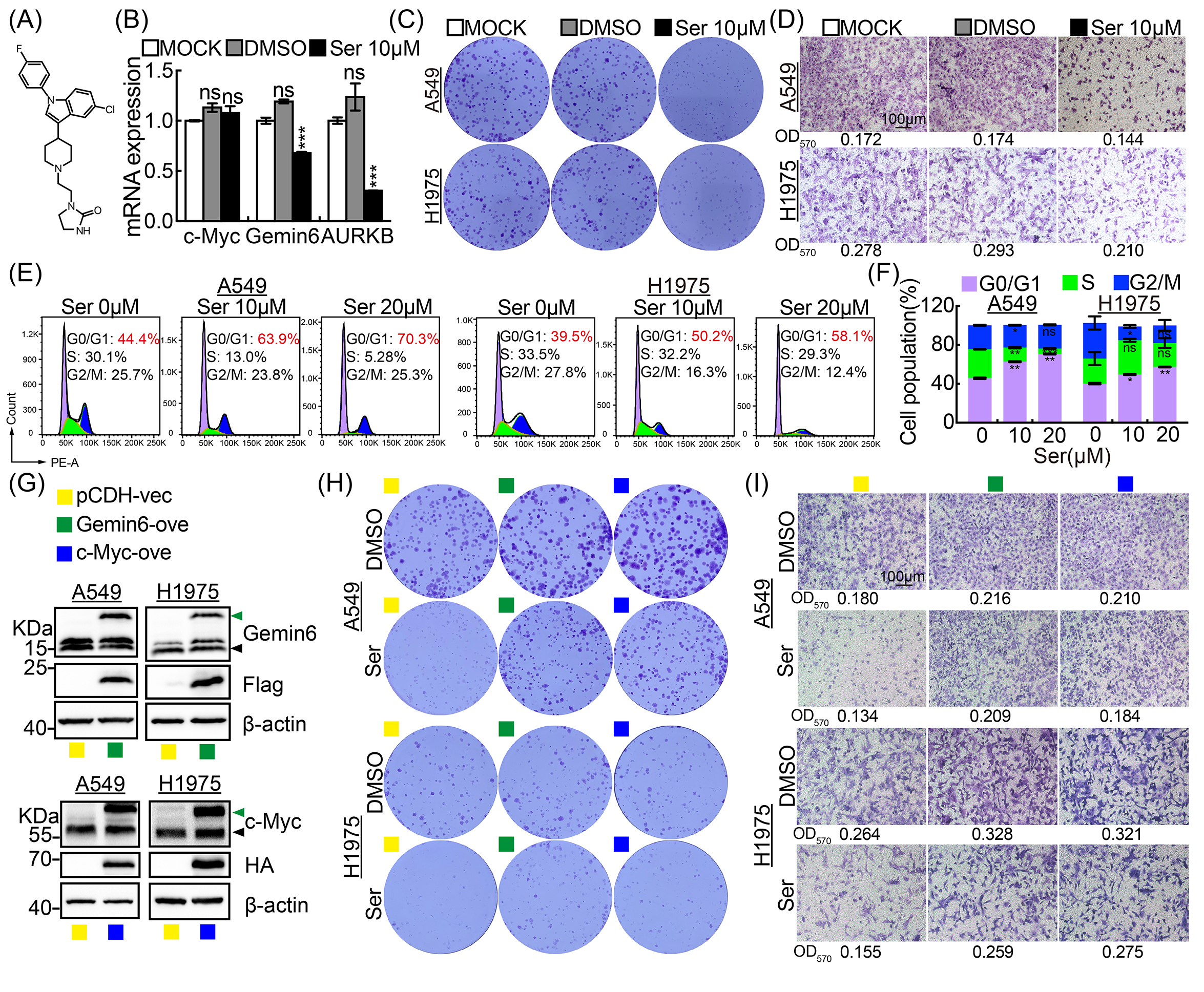
Supplementary Figure S4. Sertindole inhibits tumor cell growth and migration. (A) Molecular structural of sertindole. (B) The relative mRNA expressions of c-Myc, Gemin6 and AURKB in H1975 with or without 10 µM sertindole treatment were detected by Real-time RT-PCR. Unpaired *t*-test. (C-D) Sertindole (10 μM) treatment in A549 and H1975 cells decreased cell proliferation and migration examined by colony formation (C) and trans-well (D) assays. Scale bar: 100 μm. The OD_570_ values were shown at the bottom. (E-F) A549 and H1975 treated by indicated concentrations of sertindole were arrested at G0/G1 phase. (F) Quantification data for (E). Unpaired *t*-test. (G) The forced-expression of Gemin6 and c-Myc were validated by immunoblot in A549 and H1975 cells. Green arrow: exogenous protein; Black arrow: endogenous protein. (H) Representative images of the colony formation assay in indicated cells. (I) Representative images of the trans-well assay in indicated cells. Scale bar: 100 μm. The OD_570_ values were shown at the bottom. Bars are the mean value ± SEM. * *P* < 0.05, ** *P* < 0.01, *** *P* < 0.001, ns = no significant difference.

**Table S1. The pathological characteristics of patients with non-small cell lung cancer (NSCLC)**, and health donors.

| **Patients characteristics** | | | **No. (%)** | | |
| --- | --- | --- | --- | --- | --- |
| **NSCLC patients** | | |  | | |
| **Age(years)** | | |  | | |
| ≤50 | | | 46(28.8%) | | |
| >50 | | | 114(71.2%) | | |
| **Gender** | | |  | | |
| Male | | | 117(73.1%) | | |
| Female | | | 43(26.9%) | | |
|  | | |  | | |
| **Health donor** | | |  | | |
| **Age(years)** | | |  | | |
| ≤50 | | | 35(76%) | | |
| >50 | | | 11(24%) | | |
| **Gender** | | |  | | |
| Male | | | 29(63%) | | |
| Female | | | 17(37%) | | |
| **Prognostic information of 140 patients from the tumor tissue microarray used in Figure 1** | | | | | |
| **LUAD** | | | | | |
| **No**. | **Gemin6 score** | **Event (Live 0/Death 1)** | | **Time(months)** | **Metastasis (Yes/No)** |
| #1 | 6 | 0 | | 30 | Yes |
| #2 | 6 | 0 | | 44 | Yes |
| #3 | 6 | 0 | | 28 | Yes |
| #4 | 6 | 0 | | 12 | No |
| #5 | 6 | 0 | | 13 | Yes |
| #6 | 5.75 | 0 | | 33 | Yes |
| #7 | 5.75 | 1 | | 30 | Yes |
| #8 | 5.75 | 0 | | 32 | Yes |
| #9 | 5.75 | 0 | | 17 | Yes |
| #10 | 5.75 | 0 | | 8 | Yes |
| #11 | 5.75 | 0 | | 6 | No |
| #12 | 5.5 | 0 | | 40 | Yes |
| #13 | 5.5 | 0 | | 6 | Yes |
| #14 | 5.25 | 1 | | 28 | Yes |
| #15 | 5.25 | 1 | | 10 | Yes |
| #16 | 5.25 | 1 | | 40 | Yes |
| #17 | 5.25 | 0 | | 10 | Yes |
| #18 | 5 | 0 | | 12 | No |
| #19 | 5 | 0 | | 19 | Yes |
| #20 | 5 | 0 | | 31 | Yes |
| #21 | 5 | 0 | | 16 | Yes |
| #22 | 5 | 0 | | 33 | Yes |
| #23 | 5 | 0 | | 8 | Yes |
| #24 | 4.75 | 1 | | 24 | Yes |
| #25 | 4.75 | 0 | | 10 | Yes |
| #26 | 4.75 | 0 | | 10 | Yes |
| #27 | 4.5 | 0 | | 27 | Yes |
| #28 | 4.5 | 1 | | 8 | Yes |
| #29 | 4.5 | 1 | | 11 | Yes |
| #30 | 4.5 | 0 | | 5 | Yes |
| #31 | 4.25 | 0 | | 18 | No |
| #32 | 4.25 | 0 | | 14 | Yes |
| #33 | 4.25 | 1 | | 14 | Yes |
| #34 | 4.25 | 0 | | 8 | Yes |
| #35 | 4.25 | 1 | | 26 | Yes |
| #36 | 4.25 | 0 | | 35 | Yes |
| #37 | 4.25 | 1 | | 24 | Yes |
| #38 | 4 | 0 | | 47 | No |
| #39 | 4 | 0 | | 26 | Yes |
| #40 | 4 | 0 | | 49 | No |
| #41 | 4 | 1 | | 26 | No |
| #42 | 4 | 1 | | 36 | Yes |
| #43 | 4 | 0 | | 24 | Yes |
| #44 | 4 | 0 | | 25 | Yes |
| #45 | 4 | 1 | | 24 | Yes |
| #46 | 4 | 0 | | 26 | No |
| #47 | 4 | 0 | | 46 | Yes |
| #48 | 4 | 0 | | 21 | Yes |
| #49 | 4 | 0 | | 20 | Yes |
| #50 | 3.75 | 0 | | 52 | Yes |
| #51 | 3.75 | 0 | | 53 | Yes |
| #52 | 3.75 | 0 | | 63 | Yes |
| #53 | 3.75 | 0 | | 44 | No |
| #54 | 3.5 | 0 | | 27 | No |
| #55 | 3.5 | 0 | | 35 | Yes |
| #56 | 3.5 | 1 | | 12 | Yes |
| #57 | 3.25 | 0 | | 10 | No |
| #58 | 3 | 0 | | 62 | Yes |
| #59 | 3 | 0 | | 18 | No |
| #60 | 3 | 0 | | 62 | No |
| #61 | 3 | 0 | | 57 | Yes |
| #62 | 3 | 0 | | 29 | Yes |
| #63 | 2.75 | 0 | | 11 | No |
| #64 | 2.5 | 0 | | 14 | No |
| #65 | 2.5 | 0 | | 19 | No |
| #66 | 2.5 | 0 | | 10 | No |
| #67 | 2.5 | 0 | | 20 | No |
| #68 | 2.25 | 0 | | 58 | Yes |
| #69 | 2.25 | 0 | | 48 | No |
| #70 | 1.5 | 0 | | 14 | No |
| **LUSC** | | | | | |
| **No**. | **Gemin6 score** | **Event (Live 0/Death 1)** | | **Time(months)** | **Metastasis (Yes/No)** |
| #1 | 6 | 0 | | 15 | Yes |
| #2 | 6 | 0 | | 15 | Yes |
| #3 | 6 | 0 | | 8 | Yes |
| #4 | 6 | 0 | | 28 | Yes |
| #5 | 6 | 0 | | 25 | No |
| #6 | 6 | 0 | | 20 | Yes |
| #7 | 6 | 0 | | 23 | No |
| #8 | 6 | 0 | | 32 | Yes |
| #9 | 6 | 0 | | 7 | No |
| #10 | 6 | 0 | | 17 | Yes |
| #11 | 6 | 0 | | 11 | No |
| #12 | 6 | 0 | | 6 | Yes |
| #13 | 6 | 0 | | 6 | Yes |
| #14 | 5.75 | 0 | | 22 | Yes |
| #15 | 5.75 | 1 | | 3 | Yes |
| #16 | 5.75 | 1 | | 4 | Yes |
| #17 | 5.75 | 0 | | 27 | Yes |
| #18 | 5.5 | 1 | | 24 | Yes |
| #19 | 5.5 | 0 | | 15 | No |
| #20 | 5.5 | 0 | | 26 | Yes |
| #21 | 5.5 | 1 | | 31 | Yes |
| #22 | 5.5 | 0 | | 13 | No |
| #23 | 5.25 | 0 | | 3 | No |
| #24 | 5.25 | 0 | | 18 | No |
| #25 | 5.25 | 0 | | 19 | Yes |
| #26 | 5 | 1 | | 9 | Yes |
| #27 | 5 | 1 | | 9 | Yes |
| #28 | 5 | 0 | | 22 | Yes |
| #29 | 5 | 0 | | 29 | Yes |
| #30 | 5 | 0 | | 10 | Yes |
| #31 | 5 | 0 | | 20 | No |
| #32 | 4.75 | 1 | | 13 | No |
| #33 | 4.75 | 0 | | 30 | Yes |
| #34 | 4.75 | 0 | | 7 | Yes |
| #35 | 4.75 | 0 | | 7 | Yes |
| #36 | 4.5 | 0 | | 8 | No |
| #37 | 4.5 | 0 | | 24 | Yes |
| #38 | 4.5 | 0 | | 13 | Yes |
| #39 | 4.5 | 0 | | 9 | Yes |
| #40 | 4.5 | 0 | | 10 | No |
| #41 | 4.25 | 1 | | 4 | No |
| #42 | 4.25 | 0 | | 29 | Yes |
| #43 | 4.25 | 0 | | 23 | Yes |
| #44 | 4.25 | 0 | | 27 | Yes |
| #45 | 4.25 | 0 | | 9 | Yes |
| #46 | 4.25 | 0 | | 7 | Yes |
| #47 | 4 | 1 | | 24 | Yes |
| #48 | 4 | 0 | | 15 | Yes |
| #49 | 4 | 0 | | 22 | Yes |
| #50 | 4 | 0 | | 66 | No |
| #51 | 4 | 0 | | 53 | No |
| #52 | 4 | 0 | | 30 | Yes |
| #53 | 4 | 0 | | 47 | No |
| #54 | 4 | 0 | | 20 | No |
| #55 | 4 | 1 | | 5 | Yes |
| #56 | 4 | 0 | | 19 | No |
| #57 | 4 | 0 | | 18 | No |
| #58 | 4 | 0 | | 7 | No |
| #59 | 4 | 0 | | 27 | Yes |
| #60 | 4 | 0 | | 10 | No |
| #61 | 3.75 | 1 | | 30 | Yes |
| #62 | 3.75 | 0 | | 33 | No |
| #63 | 3.25 | 0 | | 43 | No |
| #64 | 3.25 | 0 | | 24 | Yes |
| #65 | 3 | 0 | | 33 | Yes |
| #66 | 3 | 0 | | 27 | Yes |
| #67 | 2.75 | 0 | | 21 | No |
| #68 | 2.5 | 0 | | 30 | No |
| #69 | 2 | 0 | | 48 | Yes |
| #70 | 2 | 0 | | 36 | Yes |

**Table S2. The summary of Copy Number Variation (CNV) of GEMIN6 in the pan-cancers.**

| **Cancer type** | **Symbol** | **Amp_total** | **Dele_total** | **Amp_hete** | **Dele_hete** | **Amp_homo** | **Dele_homo** | **entrez** |
| --- | --- | --- | --- | --- | --- | --- | --- | --- |
| BLCA | GEMIN6 | 31.8627451 | 7.8431373 | 30.6372549 | 7.8431373 | 1.2254902 | 0 | 79833 |
| CESC | GEMIN6 | 26.440678 | 4.0677966 | 25.4237288 | 4.0677966 | 1.0169492 | 0 | 79833 |
| CHOL | GEMIN6 | 13.8888889 | 5.5555556 | 13.8888889 | 5.5555556 | 0 | 0 | 79833 |
| COAD | GEMIN6 | 18.1818182 | 1.7738359 | 18.1818182 | 1.7738359 | 0 | 0 | 79833 |
| ESCA | GEMIN6 | 36.9565217 | 8.1521739 | 36.4130435 | 7.6086957 | 0.5434783 | 0.5434783 | 79833 |
| KICH | GEMIN6 | 3.030303 | 69.6969697 | 3.030303 | 69.6969697 | 0 | 0 | 79833 |
| KIRC | GEMIN6 | 14.3939394 | 3.030303 | 14.3939394 | 2.8409091 | 0 | 0.1893939 | 79833 |
| KIRP | GEMIN6 | 14.9305556 | 2.4305556 | 14.5833333 | 2.0833333 | 0.3472222 | 0.3472222 | 79833 |
| LIHC | GEMIN6 | 14.0540541 | 8.3783784 | 13.2432432 | 8.3783784 | 0.8108108 | 0 | 79833 |
| LUAD | GEMIN6 | 29.2635659 | 6.3953488 | 28.6821705 | 6.3953488 | 0.5813953 | 0 | 79833 |
| LUSC | GEMIN6 | 49.9001996 | 3.5928144 | 49.3013972 | 3.3932136 | 0.5988024 | 0.1996008 | 79833 |
| OV | GEMIN6 | 38.6873921 | 10.7081174 | 35.9240069 | 10.5354059 | 2.7633851 | 0.1727116 | 79833 |
| READ | GEMIN6 | 21.8181818 | 9.0909091 | 21.8181818 | 9.0909091 | 0 | 0 | 79833 |
| SKCM | GEMIN6 | 18.2561308 | 14.1689373 | 17.7111717 | 14.1689373 | 0.5449591 | 0 | 79833 |
| STAD | GEMIN6 | 19.047619 | 7.0294785 | 18.1405896 | 6.5759637 | 0.9070295 | 0.4535147 | 79833 |
| TGCT | GEMIN6 | 39.3333333 | 3.3333333 | 38.6666667 | 3.3333333 | 0.6666667 | 0 | 79833 |
| UCEC | GEMIN6 | 21.3358071 | 2.4118738 | 19.6660482 | 2.2263451 | 1.6697588 | 0.1855288 | 79833 |
| UCS | GEMIN6 | 44.6428571 | 3.5714286 | 41.0714286 | 3.5714286 | 3.5714286 | 0 | 79833 |
| UVM | GEMIN6 | 13.75 | 0 | 13.75 | 0 | 0 | 0 | 79833 |
| HNSC | GEMIN6 | 19.9233716 | 6.7049808 | 19.1570881 | 6.7049808 | 0.7662835 | 0 | 79833 |

Hete.(Heterozygous); Homo.(Homozygous); Amp.(Amplification); Dele.(Deletion)

**Table S3. Candidate drugs used for screen in Figure 4A.**

| **No.** | **Drug name** | **Targets** |
| --- | --- | --- |
| 1 | Aripiprazole | 5-HT Receptor |
| 2 | Clozapine | Dopamine Receptor |
| 3 | Paliperidone | Dopamine Receptor |
| 4 | Ziprasidone | 5-HT Receptor; Dopamine Receptor |
| 5 | Sertindole | 5-HT Receptor; Dopamine Receptor; Autophagy |
| 6 | Olanzapine | 5-HT Receptor; Autophagy; Mitophagy |
| 7 | Haloperidol | Dopamine Receptor |
| 8 | Asenapine | 5-HT Receptor; Dopamine Receptor |
| 9 | Quetiapine | 5-HT Receptor |
| 10 | Amisulpride | Dopamine Receptor |

**Table S4. Primers and antibody used in this study.**

| **Primer Name** | **Primer sequences (5'-3')** | | | | |
| --- | --- | --- | --- | --- | --- |
| Human β-actin_F | | AAGTGTGACGTGGACATCCGC | | | |
| Human β-actin_R | | CCGGACTCGTCATACTCCTGCT | | | |
| Human Gemin6_F | | GAAGAATGAGTATAAAGGATGGGTTT | | | |
| Human Gemin6_R | | TTCAAGGAAGTTCACAAGGACA | | | |
| Human c-Myc_F | | GGCTCCTGGCAAAAGGTCA | | | |
| Human c-Myc_R | | CTGCGTAGTTGTGCTGATGT | | | |
| Human USP36_F | | AGCACTTTTCCCCCAGAACTG | | | |
| Human USP36_R | | GGCTCCCAGATCTGCTGCTA | | | |
| Human USP28_F | | GGAACAGCAGCAAGATGTGA | | | |
| Human USP28_R | | GGCCGAAGGTCTCATTGTTA | | | |
| Human FBXW7_F | | CGACGCCGAATTACATCTGTC | | | |
| Human FBXW7_R | | CGTTGAAACTGGGGTTCTATCA | | | |
| Human SKP2_F | | ATGCCCCAATCTTGTCCATCT | | | |
| Human SKP2_R | | CACCGACTGAGTGATAGGTGT | | | |
| Human AURKB_F | | ATCAGCTGCGCAGAGAGATCGAAA | | | |
| Human AURKB_R | | CTGCTCGTCAAATGTGCAGCTCTT | | | |
| Human AURKB-Intron4_F | | TCCAGAAATCTGCCTCCAGTT | | | |
| Human AURKB-Intron4_R | | TCAAGTAGATCCTCCTCCGGT | | | |
| Human Gemin6-shRNA#1 | | GCCAATATTGTCCTTGTGAAC | | | |
| Human Gemin6-shRNA#2 | | AGTGTGAACATACTGATAGAA | | | |
| MSP-M-Gemin6_F | | TTAGGTTGGAGTGTAATGGTGC | | | |
| MSP-M-Gemin6_R | | ACACTTTAAAATACCGAAACGAAT | | | |
| MSP-U-Gemin6_F | | TTAGGTTGGAGTGTAATGGTGT | | | |
| MSP-U-Gemin6_R | | CACTTTAAAATACCAAAACAAAT | | | |
| Human UBE2T_F | | ATCCCTCAACATCGCAACTGT | | | |
| Human UBE2T_R | | CAGCCTCTGGTAGATTATCAAGC | | | |
| Human BIRC5_F | | AGGACCACCGCATCTCTACAT | | | |
| Human BIRC5_R | | AAGTCTGGCTCGTTCTCAGTG | | | |
| Human RNASEH2A_F | | AAGACCCTATTGGAGAGCGAG | | | |
| Human RNASEH2A_R | | AGTTCAGGTTGTATTTGACCCG | | | |
| Human NOP16_F | | GGTTACAGTGTCAACCGAAAGC | | | |
| Human NOP16_R | | GATGTGGGAGCATTCGATCCG | | | |
| Human RCF4_F | | TTGGGCCTGAACTTTTCCGAT | | | |
| Human RCF4_R | | AGCGACTTCCTGACACAGTTA | | | |
| Human CDC45_F | | TTCGTGTCCGATTTCCGCAAA | | | |
| Human CDC45_R | | TGGAACCAGCGTATATTGCAC | | | |
| **Antibody Name** | **Catalog number** | | **Dilution** | **Supplier** | **Species** |
| β-actin | 60008-1-1g | | 1:5000 | Proteintech | Mouse |
| Gemin6 | 12307-2-AP | | 1:1000 | Proteintech | Rabbit |
| CDK2 | 10122-1-AP | | 1:2000 | Proteintech | Rabbit |
| CDK4 | ab108357 | | 1:2000 | abcam | Rabbit |
| CDK6 | ab124821 | | 1:2000 | abcam | Rabbit |
| E-cadherin | ab40772 | | 1:1000 | abcam | Rabbit |
| N-cadherin | ab18203 | | 1:1000 | abcam | Rabbit |
| Vimentin | 103661-1-AP | | 1:2000 | Proteintech | Rabbit |
| Slug | 9585 | | 1:1000 | CST | Rabbit |
| c-Myc | SC-40 | | 1:1000 | Santa cruz | Mouse |
| AURKB | 3094 | | 1:1000 | CST | Rabbit |
| c-Myc^ser67^ | NA | | 1:2000 | Dr. Hudan Liu’s Lab | Rabbit |
| SMN | SC-32313 | | 1:1000 | Santa cruz | Mouse |
| Gemin7 | PA5-46733 | | 1:1000 | Invitrogen | Rabbit |
| Flag | F1804 | | 1:2000 | Sigma | Mouse |
| HA | sc-7392 | | 1:2000 | Santa cruz | Mouse |
| Ki67 | RMA-0542 | | 1:500 | MXBiotech | Rabbit |
